# Disease activity and biologic use in patients with psoriatic arthritis or rheumatoid arthritis

**DOI:** 10.1007/s10067-018-4140-0

**Published:** 2018-06-04

**Authors:** Arthur Kavanaugh, Rakesh Singh, Chitra Karki, Carol J. Etzel, Joel M. Kremer, Jeffrey D. Greenberg, Jenny Griffith

**Affiliations:** 1 University of California, San Diego, 9500 Gilman Drive, MC 0943, La Jolla, CA 92093-0943 USA; 2 AbbVie Inc., North Chicago, IL USA; 3 Corrona, LLC, Southborough, MA USA; 4 Albany Medical College, Albany, NY USA; 5 New York University School of Medicine, New York, NY USA

**Keywords:** Biologics, Disease burden, Psoriatic arthritis, Rheumatoid arthritis

## Abstract

To compare disease burden and biologic use among psoriatic arthritis (PsA) or rheumatoid arthritis (RA) patients recruited to the Corrona registry. Retrospective study of patients with PsA or RA enrolled in Corrona between January 2002 and March 2013 and grouped in 2-year intervals. Clinical outcomes and biologic use were assessed. Biologic use increased over time in both cohorts, with 62 and 52% of patients with PsA and RA, respectively, receiving biologics by 2012–2013. However, 25 and 35% of patients with PsA and RA, respectively, continued to experience moderate/high disease activity. Overall, the progressive increase in biologic use accompanied progressive decreases in Clinical Disease Activity Index (from 14.2 to 10.4 for RA, and 12.4 to 8.1 for PsA) and mean Health Assessment Questionnaire score (from 0.36 to 0.34, and 0.3 to 0.24). Mean patient pain, the proportion of patients reporting morning stiffness, and the mean duration of morning stiffness remained similar for both cohorts. PsA and RA treated in the rheumatology setting had a comparable impact on patient quality of life and functional ability. Disease burden improved with increased biologic utilization in both groups; however, moderate/severe disease remains in a significant proportion of PsA and RA patients.

## Introduction

Psoriatic arthritis (PsA) is a chronic, progressive, inflammatory arthritis, which affects 20–30% of patients with psoriasis [1]. Historically, PsA was considered to be a less severe form of inflammatory arthritis; however, it has since been demonstrated that PsA can be associated with substantial joint damage and disability [2].

Cutaneous lesions often precede the appearance of joint manifestations in PsA, so dermatologists often have the first opportunity to diagnose and treat [3]. Patients who develop joint manifestations are then typically referred to a rheumatologist [3]. One common treatment pathway is for patients to be offered initial therapy with one or more disease-modifying antirheumatic drugs (DMARDs), and if this initial treatment fails, a biologic drug is considered [4].

It is estimated that PsA-related joint erosions and destructions are similar to those of rheumatoid arthritis (RA) [2], and yet, although the clinical burden of RA is well established [5, 6], the clinical burden associated with PsA is not as adequately quantified [7]. However, evidence suggests that the clinical features of PsA can culminate in reduced physical and psychosocial health-related quality of life and an increased economic burden, with the latter resulting from direct medical costs and indirect costs due to disability and lost productivity [7–9].

In this large, real-world study, we directly compared the disease burden and use of biologic drugs between 2002 and 2013 in patients with PsA or RA who had been referred to the rheumatology setting and enrolled in the Consortium of Rheumatology Researchers of North America (Corrona) registry. The objective of this analysis was to discover whether the disease burden of PsA was different from that of RA, whether this difference had changed over time, and whether biologic treatment patterns had altered in the wake of increases in published efficacy and safety data.

## Patients and methods

### Study design and population

This was a retrospective observational cohort study of patients with PsA or RA enrolled in the Corrona registry between January 2002 and March 2013. The ongoing Corrona registry is an independent, prospective observational cohort of patients with RA or PsA, recruited from 171 private and academic practice sites across 40 states in the USA. As of April 2017, 676 rheumatologists and 45,722 patients had participated.

Corrona registry patients aged > 18 years with a PsA or RA diagnosis and an eligible (last) visit between January 1, 2002 to March 31, 2013 were included in the study. PsA patients with axial symptoms were excluded from the analysis. Patient visits during the study period were divided into 2-year intervals and the last visit (enrollment or follow-up visit) for each patient within the 2-year interval was evaluated.

### Outcomes of interest

Disease activity was measured by Clinical Disease Activity Index (CDAI), physical function was measured using modified Health Assessment Questionnaire (mHAQ), current biologic use (abatacept, adalimumab, anakinra, certolizumab, etanercept, golimumab, infliximab, tocilizumab, rituximab), and individual disease symptoms such as pain (measured using a visual analog scale (0–100)) and morning stiffness (% yes, and duration [< 1 or > 1 h]) were assessed and compared between RA and PsA cohorts.

### Statistical analysis

Descriptive statistics on patient characteristics were evaluated: means, standard deviations (quantitative variables), and percentages (categorical variables) were presented. Standardized differences were calculated to compare the distribution of patient characteristics, both quantitative and categorical, in patients with RA and PsA across each 2-year time interval. Logistic regression models were used to estimate adjusted odds ratio (OR, 95% confidence interval) to evaluate the likelihood of a patient having moderate/high disease activity (based on CDAI) and the use of biologics in patients with PsA compared to that with patients with RA, adjusting for covariates within each time period. Covariates used in the model included age (years), gender (male/female), duration of disease (years), age at disease onset, education (% with primary, high school, or college/university), insurance (% private), comorbidities, current biologic use, and patient location (by region).

## Results

### Demographics and disease characteristics

Patient demographics and disease characteristics are presented in Table 1. Compared with RA patients, patients with PsA were, on average, younger and had an earlier disease onset. A greater proportion of PsA patients had a college/university education and/or had full-time employment. Although patients with RA were predominantly female, the PsA cohort had an equal proportion of males and females.

**Table 1:** Demographics and disease characteristics of patients with PsA and RA across the study period

	2002–2003		2004–2005		2006–2007		2008–2009		2010–2011		2012–2013	
	RA	PsA	RA	PsA	RA	PsA	RA	PsA	RA	PsA	RA	PsA
	*n* = 4763	*n* = 457	*n* = 9494	*n* = 1055	*n* = 13,206	*n* = 1699	*n* = 16,645	*n* = 2163	*n* = 20,273	*n* = 2522	*n* = 20,982	*n* = 2552
Age, years	59.0 (13.4)	52.9 (13.1)	59.8 (13.4)	52.9 (12.6)	60.0 (13.6)	53.4 (12.7)	60.0 (13.7)	54.0 (12.8)	60.4 (13.4)	54.3 (12.9)	61.1 (13.1)	54.7 (12.8)
Female (%)	74.5	50.7	74.7	50.3	76.1	51.6	76.50	51.50	76.70	52.20	76.70	53.50
BMI	28.6 (6.7)	30.7 (6.9)	28.7 (6.8)	30.9 (7.1)	29.2 (7.5)	31.4 (7.8)	29.0 (6.9)	30.9 (7.1)	29.2 (7.0)	31.1 (7.1)	29.4 (7.0)	31.2 (6.9)
White (%)	89.1	92.8	89.8	95.4	87.6	93.6	87.6	94.3	87.4	93.6	87.9	92.3
College/university (%)	47.7	59.3	48.2	60.1	51.9	63.9	54.9	66.6	56.2	69.0	58.5	70.4
Full-time employment (%)	32.6	56.1	31.5	52.0	33.4	56.5	35.2	55.0	35.4	54.8	34.7	55.1
Age at disease onset	48.3 (14.6)	43.7 (14.1)	48.6 (14.7)	43.3 (14.0)	48.6 (14.9)	43.7 (14.0)	48.5 (14.8)	43.8 (13.8)	48.7 (14.5)	43.9 (13.8)	48.9 (14.4)	44.3 (13.7)
Duration of disease (years)	10.7 (9.7)	9.4 (8.9)	11.3 (9.9)	9.7 (9.2)	11.5 (10.2)	9.8 (8.9)	11.7 (10.1)	10.2 (9.0)	11.8 (10.1)	10.4 (9.2)	12.3 (10.3)	10.4 (9.2)
Swollen joint count (0–28)	4.3 (5.2)	3.2 (4.4)	4.3 (5.8)	2.6 (4.4)	3.4 (5.2)	2.0 (4.0)	2.8 (4.4)	1.6 (3.4)	2.7 (4.3)	1.7 (3.7)	2.5 (4.1)	1.6 (3.4)
Tender joint count (0–28)	4.1 (5.7)	3.5 (5.0)	3.4 (5.4)	2.7 (4.4)	3.0 (5.3)	2.3 (4.4)	2.8 (5.0)	2.2 (4.4)	3.1 (5.3)	2.5 (5.0)	3.2 (5.4)	2.5 (4.8)
Patient global assessment (VAS 0–100)	32.3 (25.5)	31.9 (25.2)	27.9 (25.3)	25.8 (24.2)	29.1 (25.6)	27.0 (24.4)	29.3 (26.3)	26.0 (24.1)	29.1 (26.5)	26.8 (25.7)	29.8 (26.5)	26.5 (25.2)
Physician global assessment (VAS 0–100)	26.5 (21.1)	25.5 (20.6)	21.0 (19.6)	18.7 (18.3)	17.7 (18.1)	15.1 (15.5)	17.0 (18.2)	14.3 (15.9)	17.6 (18.9)	15.0 (16.8)	17.6 (18.8)	14.3 (16.5)
Erosive disease, yes (%)	57.2	52.4	53.2	42.7	53.6	40.9	52.7	39.6	49.8	38.1	44.6	35.1

Data are mean and SD unless otherwise specified

*BMI*, body mass index; *PsA*, psoriatic arthritis; *RA*, rheumatoid arthritis; *VAS*, visual analog scale

### Disease activity, disability, and biologics use

**Fig. 1:**
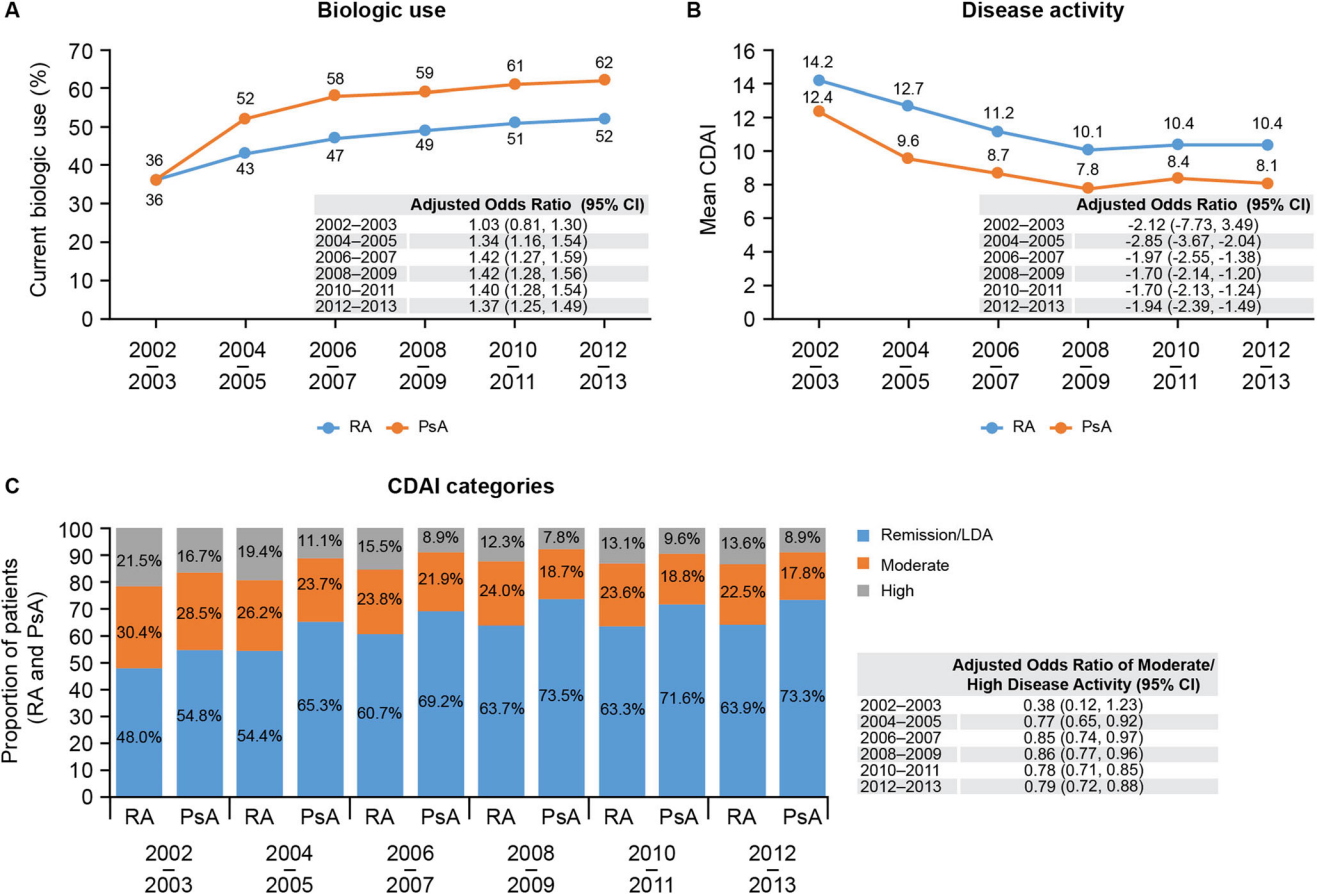
Current biologic use (**a**), mean disease activity (**b**), and disease activity by CDAI categories (**c**) in patients with PsA and RA across the study period. Covariates used in the models were age, gender, duration of disease, age of disease onset, education, insurance, comorbidities, current biologic use (except for **a**) and region of country. CI, confidence interval; CDAI, clinical disease activity index; PsA, psoriatic arthritis; RA, rheumatoid arthritis

Over the study period, the use of biologics increased in both the PsA and RA cohorts, although over time, PsA patients were > 30% more likely to receive a biologic than their RA counterparts (OR range from 1.03 to 1.42). By 2012–2013, 62% of patients with PsA were receiving a biologic compared with 52% of RA patients (Fig. 1a). The progressive increase in biologic use was accompanied by a similar progressive decrease in overall disease activity over time. The mean CDAI decreased from 12.4 to 8.1 in the PsA cohort, and from 14.2 to 10.4 in the RA cohort; while disease activity in the patients with PsA remained slightly lower than that of their RA counterparts (ORs < 1.0 across all time points) (Fig. 1b). Despite these improvements, 25 and 35% of patients with PsA and RA, respectively, continued to experience moderate or high disease activity (CDAI > 10) in the last 2-year interval (2012–2013) analyzed (Fig. 1c).

**Fig. 2:**
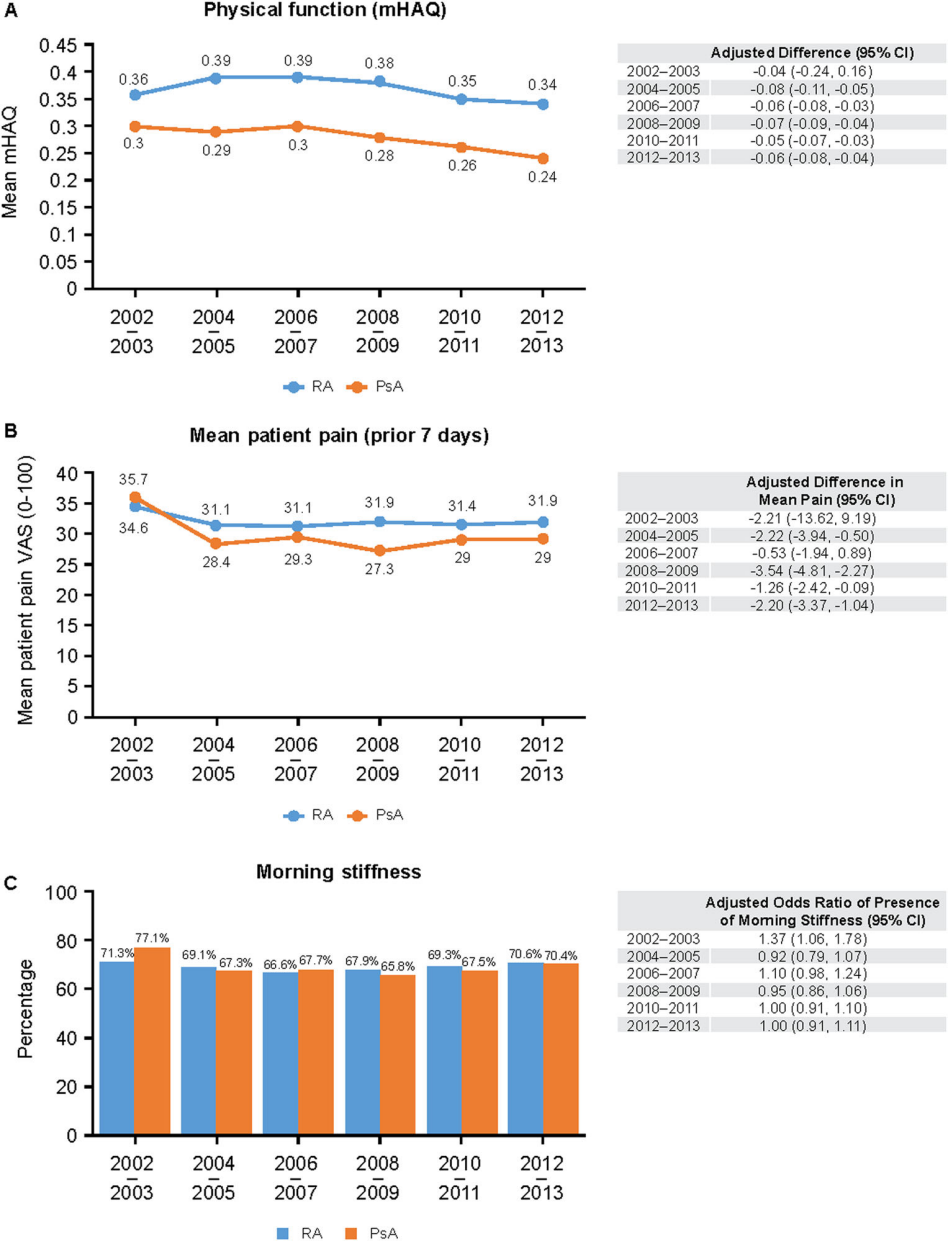
Physical function (**a**), patient pain (**b**), and morning stiffness (**c**) in patients with PsA and RA across the study period. Covariates used in the models were age, gender, duration of disease, age of disease onset, education, insurance, comorbidities, current biologic use, and region of country. CI, confidence interval; mHAQ, modified Health Assessment Questionnaire; PsA, psoriatic arthritis; RA, rheumatoid arthritis; VAS, visual analog scale

The progressive increase in biologic use and decrease in disease activity was accompanied by a similar progressive decrease in physical function, with the mean mHAQ in PsA patients being slightly lower than that of the RA patients over time (Fig. 2a). Overall, the mean mHAQ decreased from 0.30 to 0.24 in the PsA cohort, and 0.36 to 0.34 in the RA cohort. However, other patient-reported outcomes such as mean patient pain (Fig. 2b), the proportion of patients reporting morning stiffness (Fig. 2c), and the mean duration of morning stiffness (data not shown) remained similar between RA and PsA patients for the duration of the study.

## Discussion

The disease burden associated with RA is well established [5, 6]; the PsA-related burden less so. Our data show that PsA and RA patients, when treated in the rheumatologist’s office, show comparable improvements in patient quality of life and functional ability. Although joint burden, as measured by mean CDAI, was slightly lower in PsA patients than that in RA patients, rheumatologists utilized biologic agents more frequently among the PsA patients.

Our results show that over the study period, 2002 until 2013, there was an increase in the use of biologic drugs. Over this same time period, there was a progressive decrease in disease burden for both RA and PsA. However, by the end of the study, about a quarter of patients with PsA and one third of patients with RA continued to experience moderate/high disease activity.

A delay of 1 year between symptom onset and diagnosis has been associated with poorer physical function in established PsA [10], while clinical and radiographic damage have been shown to be more marked in patients presenting with > 2 years of disease duration before treatment is initiated [11, 12]. Peripheral joint erosions and worse functional outcomes have also been demonstrated with a delay as short as 6 months from symptom onset to the first rheumatology visit [12]. Therefore, early diagnosis and effective treatment of PsA may help prevent long-term joint damage and disability.

Some current treatment recommendations for PsA suggest treatment with anti-inflammatory drugs and a combination of one or more DMARDs before administering biologic drugs [4]. However, the efficacy of DMARDs has not been proven across all of the diverse manifestations of the disease. Traditional DMARDs have been shown to be ineffective for the axial manifestations of ankylosing spondylitis, and there are no data to suggest axial symptoms in PsA patients would be effectively treated by such agents [13]. For other manifestations, such as enthesitis and dactylitis, evidence supporting the clinical efficacy of targeted biological therapies in PsA is much more robust than that for traditional therapies [13, 14].

Our study had some limitations. Corrona is an observational registry and its data were restricted to information captured routinely by US physicians in regular clinical practice (e.g., CDAI was evaluated as laboratory measures, a key component of other composite indexes used in clinical trials that are not routinely collected). Additionally, as CDAI is based on a 28-joint count, additional disease impact on joints beyond those evaluated are not represented. Our registry data were also restricted to assessing the impact of PsA on joints; so despite measurements being more widespread now, data on skin involvement and impact of extra-articular manifestations were not investigated during the study period. Additionally, the data are from a rheumatology setting, so there may be referral bias in that PsA patients who require biologics are more likely to be referred here; there may well be a larger percentage of untreated patients in community practices. As with all registries, the characteristics of the enrolled patient population may not be entirely representative of the entire patient population. These hypotheses were partly assessed in a linkage study by Curtis et al., which matched 30,000 records from RA patients in the Corrona registry with their corresponding Medicare data. As hypothesized above, the authors found that the Corrona patients were more likely to take DMARDs and biologic agents, but that other than this difference, the demographics and comorbidity profiles of these registry patients were generalizable to the US Medicare population [15].

Our study assessment period ended in March 2013. Future research should focus on evolving trends in the treatment of PsA since this time and should directly compare the safety and efficacy of other RA/PsA medications in both the dermatology and rheumatology settings.

## Conclusions

The overall disease burden of PsA remains slightly lower than that of RA, but some joint-related symptoms (i.e., pain, morning stiffness) are comparable. The PsA disease burden reported in the rheumatology setting has decreased during the past decade, and this appears to have coincided with an increased use of biologic treatments. However, there continues to be a substantial proportion of PsA patients with moderate/high disease activity. Therefore, earlier and more targeted treatment of PsA joint symptoms may help to improve long-term outcomes.
